# Comprehensive analysis of m6A modification in immune infiltration, metabolism and drug resistance in hepatocellular carcinoma

**DOI:** 10.1186/s12935-024-03307-3

**Published:** 2024-04-16

**Authors:** Yunxing Shi, Kai Li, Yichuan Yuan, Chenwei Wang, Zhiwen Yang, Dinglan Zuo, Yi Niu, Jiliang Qiu, Binkui Li, Yunfei Yuan, Wei He

**Affiliations:** 1 Department of General Surgery (Colorectal Surgery), The Sixth Affiliated Hospital, Sun Yat- sen University, Guangzhou, China; 2 Guangdong Provincial Key Laboratory of Colorectal and Pelvic Floor Diseases, The Sixth Affiliated Hospital, Sun Yat-sen University, Guangzhou, China; 3 Department of Hepatobiliary Surgery, The First Affiliated Hospital of Guangzhou Medical University, Guangzhou, China; 4 State Key Laboratory of Oncology in South China and Collaborative Innovation Center for Cancer Medicine, Sun Yat-Sen University Cancer Center, Guangzhou, China; 5 Department of Liver Surgery, Sun Yat-sen University Cancer Center, 651 Dongfeng Road E, 510060 Guangzhou, P.R. China

**Keywords:** M6A modification, Tumor immune microenvironment, Metabolism, Targeted-therapy, Immunotherapy, Drug resistance

## Abstract

N6-methyladenosine (m6A) is important in regulating mRNA stability, splicing, and translation, and it also contributes to tumor development. However, there is still limited understanding of the comprehensive effects of m6A modification patterns on the tumor immune microenvironment, metabolism, and drug resistance in hepatocellular carcinoma (HCC). In this study, we utilized unsupervised clustering based on the expression of 23 m6A regulators to identify m6A clusters. We identified differential m6A modification patterns and characterized m6A-gene-cluster A, which exhibited poorer survival rates, a higher abundance of Treg cells, and increased expression of TGFβ in the tumor microenvironment (TME). Additionally, m6A-gene-cluster A demonstrated higher levels of glycolysis activity, cholesterol metabolism, and fatty acid biosynthesis. We also found that the m6A score was associated with prognosis and drug resistance. Patients with a low m6A score experienced worse prognoses, which were linked to an abundance of Treg cells, upregulation of TGFβ, and increased metabolic activity. HCC patients with a higher m6A score showed improved prognosis following sorafenib treatment and immunotherapy. In conclusion, we reveals the association between m6A modification patterns and the tumor immune microenvironment, metabolism, and drug resistance in HCC. Furthermore, the m6A score holds potential as a predictive factor for the efficacy of targeted therapy and immunotherapy in HCC.

**Supplementary Information:**

The online version contains supplementary material available at 10.1186/s12935-024-03307-3.

## Introduction

Hepatocellular carcinoma (HCC) ranks as the third leading cause of cancer-related mortality in the world [1]. Most HCC patients are diagnosed at advanced stages, resulting in poor prognosis. Interventional therapy, targeted therapy and immunotherapy are main treatments options available for those patients [2]. Sorafenib and lenvatinib have been approved as first-line targeted treatment of advanced stage HCC [3, 4]. The recent success of immune checkpoint inhibitors to treat unresectable HCC has raised interest in investigating antitumor immunity [5]. Atezolizumab plus Bevacizumab was also approved for the first-line targeted treatment of advanced stage HCC [6]. However, due to the unsatisfactory response rate, it is crucial to understand the mechanisms and predict resistance to targeted therapy or immunotherapy.

The development and progression of hepatocellular carcinoma (HCC) involve complex processes with genetic, epigenetic, and transcriptomic alterations [7]. Epigenetics changes, including DNA methylation, histone modification, and RNA-mediated targeting, have the potential to contribute to cancer progression. One common post-transcriptional modification found in messenger RNA (mRNA) is N6-methyladenosine (m6A) modification [8]. A total of 3 functional categories of protein participates in the m6A modification: methyltransferases (“writers”, METTL3/14, WTAP, RBM15/15B, ZC3H13, CBLL1 and KIAA1429), demethylases (“erasers”, FTO, ALKBH3/5) and effector proteins (“readers”, YTHDF1/2/3, YTHDC1/2, IGF2BP1/2/3, HNRNPC, ELAVL1, EIF3 and HNRNPA2B1) [9–11]. M6A modification plays vital roles in biological process across different cancer types. For instance, WTAP suppressed ETS1 in a post-transcriptional, promoting HCC progress [12]. METTL3 facilitated SOCS2 mRNA degradation through a YTHDF2-dependent pathway in HCC [7]. In non-small cell lung cancer (NSCLC), METTL3 directly promotes YAP translation and increases YAP activity by regulating the MALAT1-miR-1914-3p-YAP axis, leading to drug resistance and metastasis [13]. M6A-dependent glycolysis enhances colorectal cancer progression [14]. Furthermore, m6A modification also regulates signaling pathways involved in targeted-therapy resistance, such as AKT activity [15], EGFR signaling pathway [16], WNT signaling and stemness [17]. However, the correlation between m6A modification patterns and targeted-therapy resistance in HCC remains poorly understood.

M6A can regulate immune response to viruses and exhibit crucial impact on immune microenvironment in various cancers by controlling signal transduction [18–20]. METTL3 and YTHDF1 have been found to enhance cross-presentation of tumor antigens and stimulating CD8 + T cells through the regulation of dendritic cells activation and T cell homeostasis [21–23]. However, METTL3 also sustains the function of Treg cells to inhibit immune response [24]. The regulation of m6A in immunity appears to be complex. There have been reports that FTO, an alpha-ketoglutarate dependent dioxygenase, induced resistance to aiti-PD-1 therapy in melanoma [25]. Additionally, m6A modification patterns have shown efficacy in predicting the response and outcome of anti-PD-1/L1 therapy in gastric cancer [26]. However, the effect of m6A regulators and m6A modification on immunotherapy in HCC have not been described.

M6A regulators have also been involved in metabolic processes. METTL3 and IGF2BP2 promote glycolysis and tumorigenesis in colorectal cancer and gastric cancer [14, 27, 28]. METTL3 has also been identified as a regulator of fatty acid metabolism [29]. However, a comprehensive analysis for the functions of m6A regulators in metabolism remains scarce in HCC development.

In this study, we analyzed the landscape of genetic and expression variation of m6A regulators in HCC. Through unsupervised clustering based on the expression of 23 m6A regulators, we identified distinct m6A modification patterns in HCC. We then compared the characteristics of these patterns and discovered correlations between m6A regulators, tumor immune microenvironment and metabolism. Furthermore, we developed an m6A score based on differentially expressed genes (DEGs) to predict prognosis of HCC. The m6A score was also found to be closely associated with treatment response of sorafenib and immunotherapy resistance.

## Materials and methods

### Sample data collection and processing

TCGA data (TCGA-LIHC, 372 samples), mutations, gene expression, clinical annotations were downloaded from the TCGA data portal (https://portal.gdc.cancer.gov/) in April 2020. ICGC data (LIRI-JP), gene expression and clinical annotations were downloaded from the ICGC data portal (https://dcc.icgc.org/) in April 2020. GEO data (GSE14520, GSE76427) was available in the Gene Expression Omnibus (GEO) database. We combined TCGA-LIHC, GSE76427, ICGC-LIRI-JP data to obtain a larger cohort (669 HCCs, 292 normal). Batch effects from non-biological technical biases were corrected using the “ComBat” algorithm of sva package. All expression data was normalized using R (version 3.6.3). The somatic mutation data was acquired from cBioPortal FOR CANCER GENOMICS (https://www.cbioportal.org/) and Copy Number Variation (CNV) information was obtained from TCGA Copy Number Portal (TCGA) (http://portals.broadinstitute.org/tcga/home).

### Unsupervised clustering for 23 m6A regulators

A total of 23 regulators were extracted for identifying different m6A modification patterns mediated by m6A regulators. These 23 m6A regulators included 8 writers (METTL3, METTL14, RBM15, RBM15B, WTAP, KIAA1429, CBLL1, ZC3H13), 3 erasers (ALKBH5, ALKBH3, FTO) and 12 readers (YTHDC1, YTHDC2, YTHDF1, YTHDF2, YTHDF3, IGF2BP1, HNRNPA2B1, HNRNPC, IGF2BP2, IGF2BP3, LRPPRC, ELAVL1). Unsupervised clustering analysis was applied to identify distinct m6A modification patterns based on the expression of 23 m6A regulators and classify patients for further analysis. The number of clusters and their stability were determined by the consensus clustering algorithm [30]. We used the ConsensusClusterPlus package to perform the above steps and 1000 times repetitions were conducted for guaranteeing the stability of classification [31]. 

### Estimation of the abundance of immune cell populations, estimate score and cytolytic activity

The relative abundance of 24 immune populations in tumors and healthy tissues were computed from the RNA-seq of each bulk sample. In detail, we used the ImmuCellAI [32] a unique method for comprehensive T-cell subsets abundance prediction based on the enrichment score of gene signature, which was calculated using the single sample gene set enrichment analysis (ssGSEA) algorithm. The estimate score and tumor purity were calculated using “Estimate” [33], a method that uses gene expression signatures to infer the fraction of stromal and immune cells in tumor samples. Immune cytolytic activity representing the geometric mean of GZMA and PRF1 is another in silico measure of immune infiltration, as described by Rooney et al. [34].

### GSEA (Gene Set Enrichment Analysis), Identification of DEGs (Differentially Expressed Genes), GO (Gene Ontology) analysis and PPI (protein-protein interaction) network construction

GSEA was used to identify the pathways that were significantly enriched between m6Aclusters [35]. The GSEA analysis was performed using the GSEA software. We divided all the HCC patients into m6A cluster A and m6A cluster B and performed the GSEA using their gene expression matrix. DEGs were identified using “Limma” package in R (adjust < 0.05, |LogFC|>1) [36]. Gene Ontology (GO) analysis of DEGs was performed in WEB-based Gene Set Analysis Toolkit [37]. The STRING database was applied to get the Protein–protein interaction information [38]. A Protein–protein interaction network (PPI) was built via Cytoscape software [39]. The most significant clusters of PPI network were identified by “MCODE” and hub genes were ranked by degree. The GO analysis of hub genes was performed with “ClueGO”.

### Identification of m6A-gene-clusters and m6A score construction

The patients were classified into several groups for deeper analysis by adopting unsupervised clustering method based on DEGs. The consensus clustering algorithm was utilized for defining the number of gene clusters as well as their stability. Then, we performed the prognostic analysis for each gene in the signature using univariate Cox regression model. The genes with the significant prognosis were extracted for further analysis. We then conducted principal component analysis (PCA) to construct m6Ascore. This method had advantage of focusing the score on the set with the.

largest block of well correlated (or anticorrelated) genes in the set, while down-weighting contributions from genes that do not track with other set members. Firstly, we identified prognostic factors to construct the m6A score. A total of 50 survival-related genes were identified by univariate cox analysis by univariate analysis. Then, principal component analysis was performed using SPSS software (version 25.0) to calculate the variance contribution rate for each gene. The m6A score = Gene1.V1 + Gene2.V2……+Gene50.V50.

### Gene sets of several biological processes

Gene sets of biological processes were downloaded from MSigDB database; [40] (1) Hypoxia; (2) Glycolysis-gluconeogenesis; (3) Tricarboxylic acid cycle enzyme complex; (4) Nuclear receptors in lipid metabolism and toxicity; (5) cholesterol metabolism; (6) Regulation of fatty acid oxidation; (7) Regulation of fatty acid biosynthetic process; (8) EGF Signaling Pathway; (9) Erk1/Erk2 MAPK signaling pathway; (10) PDGF signaling pathway; (11) PI3K pathway; (12) RTK signaling; (13) Immune checkpoints; (14) Genes related to induction of pluripotent stem cells [41]. 

### mRNAsi mining

The one-class logistic regression machine learning algorithm (OCLR) was applied to extract gene expression-based stemness indices [42]. We obtained the data for the calculated mRNAsi and EREG-mRNAsi of each TCGA-LIHC patient from supplementary materials of Tathiane M. Malta’s article [42]. 

### Statistical analysis

Associations between 23 m6A regulators, DEGs and survival were tested using univariate cox regression and the hazard ratios (HR) were calculated. The median expression of each gene was used as the cutoff criteria to analyze the association between m6A regulators’ expression and overall survival. Kaplan‑Meier survival analysis by log-rank [43] test was used to calculate the median survival time (MST). The cut-off points were determined by the X-Tile software [44] to tested all potential cut points in order for finding the maximum rank statistic. Correlations among immune cell subsets, immune checkpoints and gene expression data were evaluated using the Spearman correlation coefficient. One-way ANOVA and Kruskal-Wallis tests were used to conduct difference comparisons of three or more groups [45]. Box plots for continuous variables were compared by unpaired t‑test and Mann-Whitney U test. PCA (Principal Components Analysis) was performed with SPSS or R (version 3.6.5). All statistical *P* value were two-side, with *P* < 0.05 as statistically significance. Statistical analysis was performed with GraphPad version 7.0 (GraphPad Software, Inc., La Jolla, CA, USA), SPSS software version 25.0 (SPPS, Inc., Chicago, IL, USA), R (version 3.6.5; www.r-project.org) and OriginPro 2020 (https://www.originlab.com/).

## Results

### Landscape of genetic and expression variation of m6A regulators in hepatocellular carcinoma

**Fig. 1:**
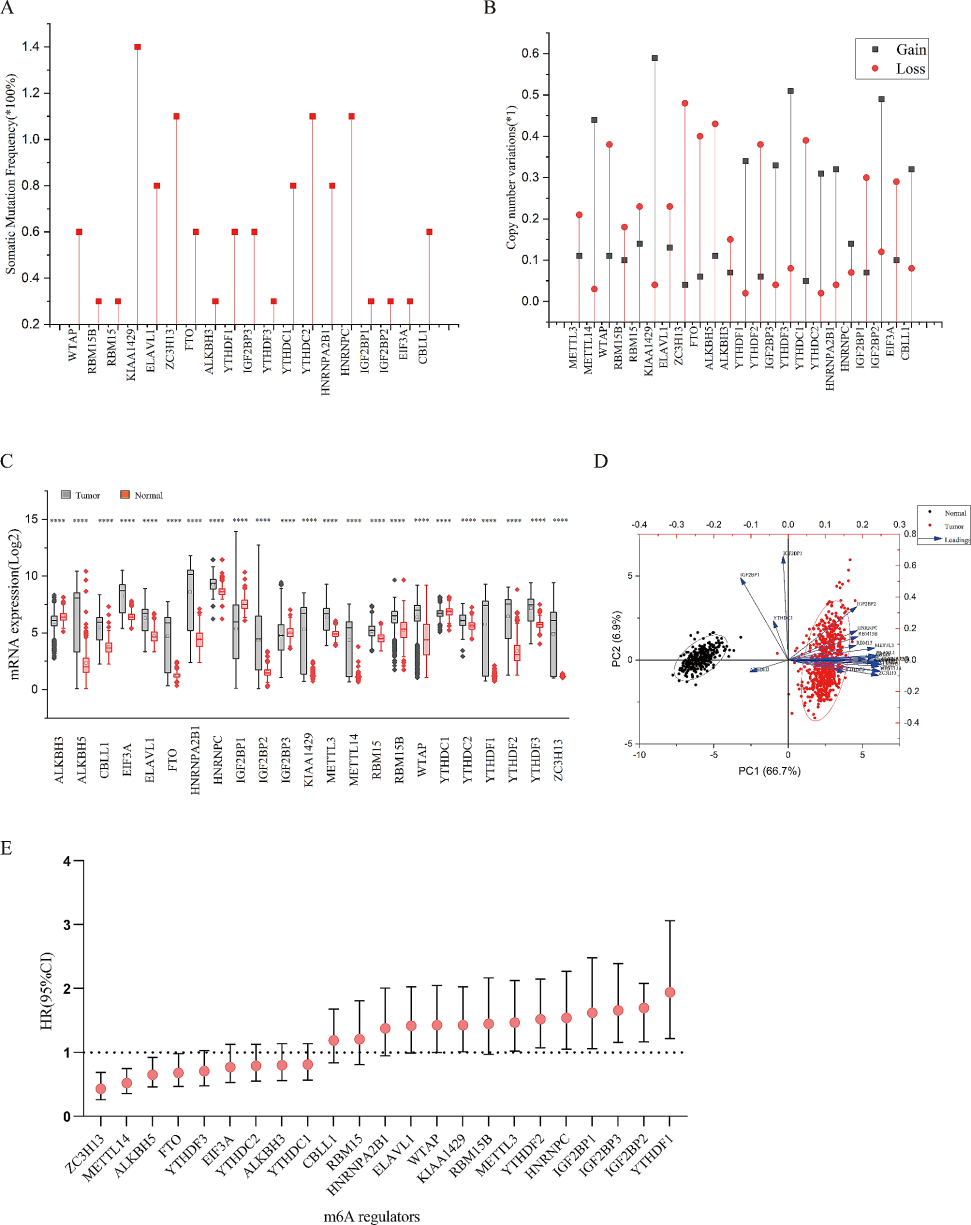
Landscape of genetic and expression variation of m6A regulators in hepatocellular carcinoma. (**A**) The mutation frequency of m6A regulators in 375 patients with hepatocellular carcinoma from TCGA-LIHC cohort. (**B**) The CNV variation frequency of m6A regulators in TCGA-LIHC cohort. The height of the column represented the alteration frequency. The deletion frequency, red dot; The amplification frequency, grey dot. (**C**) The differential expression of 23 m6A regulators between normal tissues and hepatocellular carcinoma tissues. Tumor, Grey; Normal, Red. The upper and lower ends of the boxes represented interquartile range of values. The lines in the boxes represented median value, and black dots showed outliers. The asterisks represented the statistical *p* value (*P < 0.05; **P < 0.01; ***P < 0.001; ****P < 0.0001). (**D**) Principal component analysis for the expression profiles of 23 m6A regulators to distinguish tumors from normal samples. The tumors and normal samples were well distinguished based on the expression profiles of m6A regulators. Tumors, red and normal, black. (**E**) HRs (boxes) and 95% confidence intervals (horizontal lines). Box size is inversely proportional to the width of the confidence interval

We summarized mutations and CNVs (Copy number variations) to explore genetic characteristics of m6A regulators. Among 23 m6A regulators, 19 showed somatic mutation in TCGA-LIHC cohort (Fig. 1A). KIAA1429 had the highest mutation frequency (1.4%), followed by ZC3H13(1.1%), YTHDC2(1.1%) and HNRNPC (1.1%). METTL3, METTL14, YTHDF2 and ALKBH5 did not show any mutation in database. Figure 1B showed a prevalent CNV alteration in 23 regulators. In addition, KIAA1429, YTHDF3 and IGF2BP2 had the highest frequency of CNV amplification (over 45%). Considering that m6A regulators play a vital role in cancer development [15, 46, 47], we compared their mRNA expression level in tumor and normal tissue. Of the 23 m6A regulators, 20 were significantly upregulated, while only 3 genes (ALKBH3, IGF2BP1 and YTHDC1) were downregulated in HCC (Fig. 1C). Based on the differential expression characteristics, tumor and normal samples were well distinguished based on the expression profiles of m6A regulators in principal component analysis (Fig. 1D). Furthermore, we investigated the association between the expression of m6A regulators and overall survival. Ten regulators were associated with worse overall survival, while five regulators were associated with better overall survival. (Fig. 1E).

### Correlation among 23 m6A regulators and identification of m6Aclusters

**Fig. 2:**
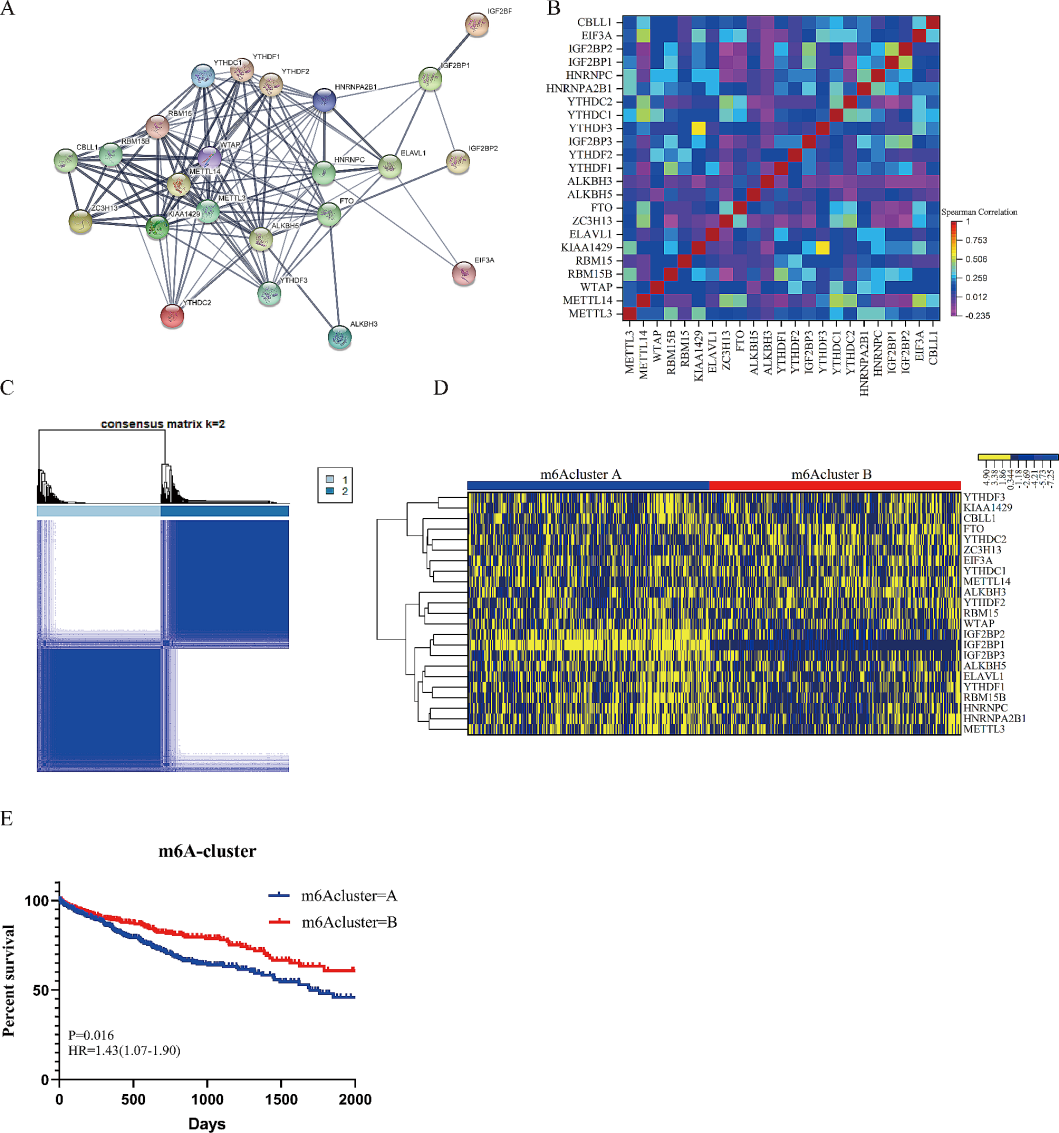
Correlation among 23 m6A regulators and identification of m6Aclusters. (**A**) Protein-protein interaction network of 23 m6A regulators. Network nodes represent proteins. Colored nodes: query proteins and first shell of interactors. White nodes: second shell of interactors. Filled nodes: some 3D structure is known or predicted. Edges represent protein-protein associations. (**B**) Correlation heatmap of m6A regulators. Different color represents spearman correlation r value between m6A regulators. (**C**) Consensus clustering analysis identification of two clusters (samples, *n* = 669). The white (consensus value = 0, samples never clustered together) and blue (consensus value = 1, samples always clustered together) heatmap display sample consensus. (**D**) heatmap of unsupervised clustering of 23 m6A regulators. Yellow represented high expression of regulators and blue represented low expression. (**E**) Survival plot of m6Aclusters (m6AclusterA:328 HCCs; m6AclusterB: 341 HCCs). The m6Acluster B showed better overall survival than m6Acluster A (*P* = 0.016; HR = 1.43)

We obtained protein-protein interaction network from STRING [38], and found complicated correlation among 23 m6A regulators (Fig. 2A). Then, we performed spearman correlation analysis based on mRNA expression and found the majority of m6A regulators significantly correlated with each other (Fig. 2B, Table S1). The GO analysis demonstrated that function of m6A regulators enriched in DNA and RNA modification (Figure S1A. These processes included the regulation of alternative mRNA, RNA stabilization, DNA dealkylation, DNA repair, oxidative demethylation. Importantly, the m6A regulators not only participated in the same biological process, but also regulated each other. KIAA1429 (“writer”), showed the strongest correlation with YTHDF3 (“reader”) (Spearman *r* = 0.62, *P* < 0.0001), indicating important cross-talk among different functional categories (“writers”, “readers”, “erasers”).

Using consensus clustering analysis Based on the mRNA expression pattern of 23 m6A regulators of 699 HCC patients, we identified 328 patients in cluster A and 341 patients in cluster B (Fig. 2C, Figure S1B-S1D). The expression patterns of 23 m6A regulators were significantly different between clusters (Fig. 2D). The m6Acluster A was prominent with higher expression of ELAVL1, HNRNPA2B1, HNRNPC, IGF2BP1, IGF2BP2, IGF2BP3, KIAA1429, METTL3, RBM15B and YTHDF1 (Figure S1E). Patients in m6Acluster B had a better overall survival compared to those in m6Acluster A (*P* = 0.016; HR = 1.43 (1.07–1.90)) (Fig. 2E).

GSEA analysis shown that differential pathways were all enriched in metabolic processes (Figure S1F), including glucose metabolism, fatty acid metabolism, retinol metabolism and ABC transporters. In addition, pathways related to drug metabolism and steroid hormone metabolism were significantly upregulated in m6Acluster B.

### Pathway enrichment of DEGs and identification of m6A-gene-clusters

**Fig. 3:**
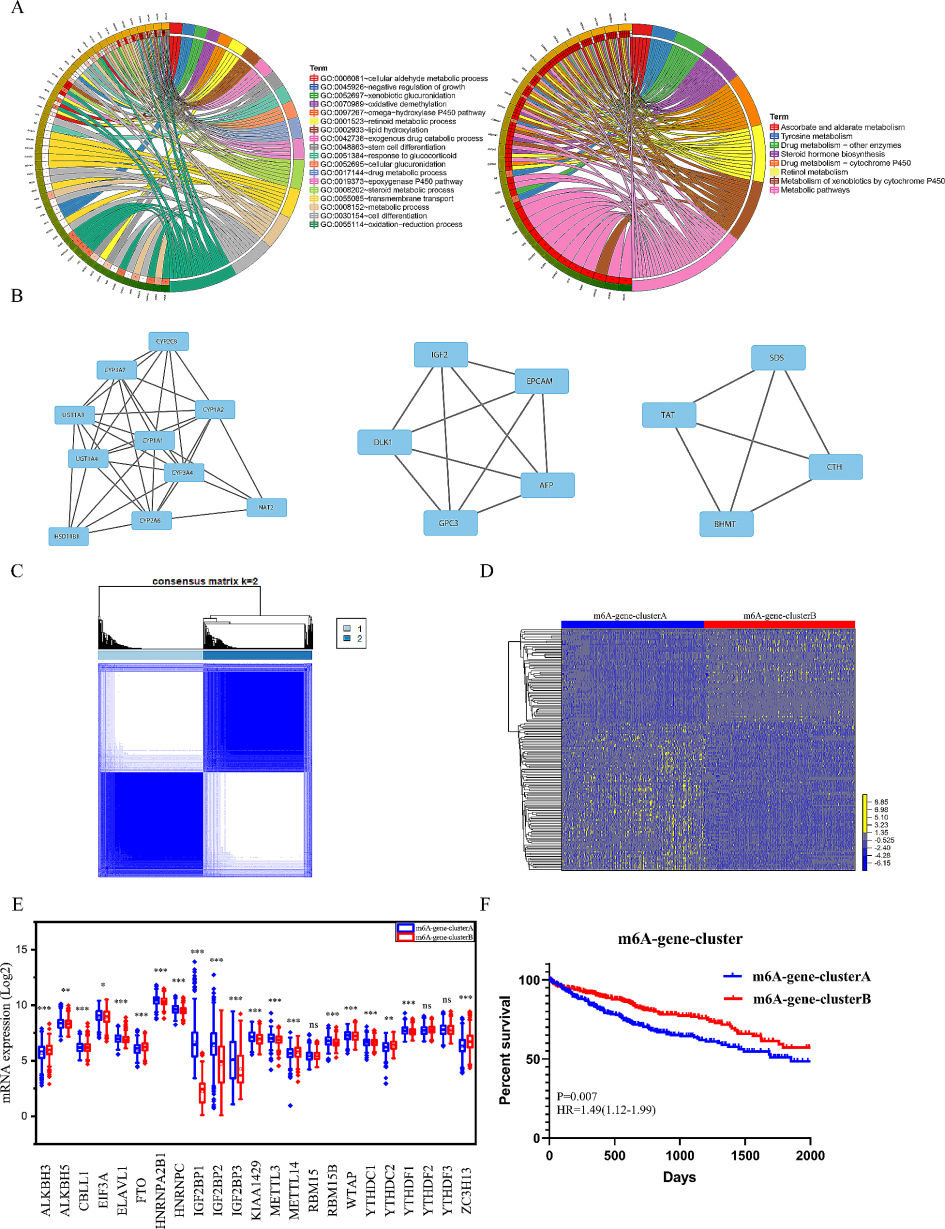
Pathway enrichment of DEGs and identification of m6A-gene-clusters. (**A**) Circo-plot of KEGG and GO analysis of 147 DEGs. Left: genes; Right: pathways. Different colors represent different pathways. (**B**) 3 significant clusters in protein-protein interaction network of 147 DEGs. Rectangles represent protein. Edges represent protein-protein associations. (**C**) Consensus clustering analysis identification of two clusters (samples, *n* = 669). The white (consensus value = 0, samples never clustered together) and blue (consensus value = 1, samples always clustered together) heatmap display sample consensus. (**D**) heatmap of unsupervised clustering of 147 DEGs. Yellow represented high expression of regulators and blue represented low expression. (**E**) The differential expression of 23 m6A regulators between m6A-gene-clusters. m6A-gene-clusterA, Grey; m6A-gene-clusterB, Red. The upper and lower ends of the boxes represented interquartile range of values. The lines in the boxes represented median value, and black dots showed outliers. (**F**) Survival plot of m6A-gene-clusters (m6A-gene-clusterA:337 HCCs; m6A-gene-clusterB: 332 HCCs). The m6A-gene-clusterB showed better overall survival than m6A-gene-clusterA (*P* = 0.007; HR = 1.49). (*P < 0.05; **P < 0.01; ***P < 0.001; ****P < 0.0001)

A total of 147 DEGs were identified between m6Acluster A and B (*P* < 0.05, FDR < 0.01) (Table S2). We performed GO and KEGG analysis to enrich pathways that m6A-related genes involved in. Consistent with results of GSEA between m6Acluster A and B, 147 DEGs were significantly involved in metabolism, including steroid metabolism, retinol metabolism, glucose metabolism and Tyrosine metabolism (Fig. 3A). Enrichment in growth and stem cell differentiation was also showed in Go analysis. Then we constructed PPI (protein-protein interaction) network (Figure S4A) and identified the most significant modules (Fig. 3B). In addition, 12 hub genes ranked by degrees were obtained and were shown in Figure S4B. These hub genes were also involved in metabolic pathways (Figure S4C).

Two m6A-gene-clusters were identified based on expression of 147 DEGs by Consensus clustering analysis (Fig. 3C and D). The prominent differences in the expression of m6A regulators between m6A-gene-clusters was in accordance with the expected results of m6A methylation modification patterns, indicating that 147 DEGs were associated with m6A modification (Fig. 3E). M6A-gene-clusterB had a better prognosis than m6A-gene-clusterB (*P* = 0.0007, HR = 1.49) (Fig. 3F).

### Differential metabolic characteristics in distinct m6A modification patterns

**Fig. 4:**
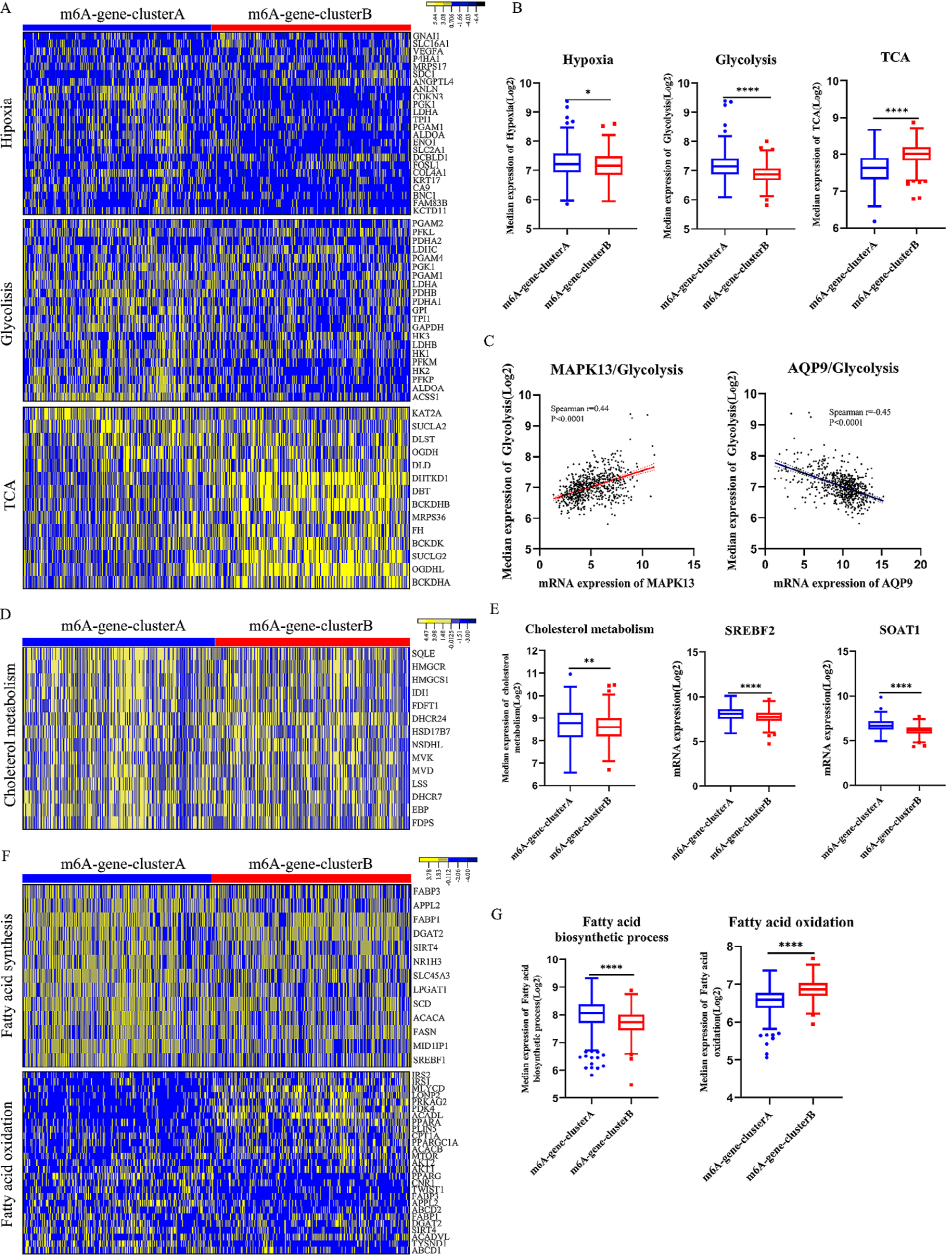
Differential metabolic characteristics in distinct m6A modification patterns. (**A**, **B**) Comparison of hypoxia and glucose metabolism related genes between clusters (**A**: heatmap; Yellow represented high expression of regulators and blue represented low expression; **B**: Median expression of genes). (**C**) Spearman correlation between IGF2BP3 and glycolytic genes expression or FBP1 expression. *P* < 0.0001. (**D, E**) Comparison of cholesterol metabolism related genes between clusters (**D**: heatmap; Yellow represented high expression of regulators and blue represented low expression; **E**: Median expression of genes). (**F, G**) Comparison of fatty acid biosynthetic process and fatty acid oxidation related genes between clusters (**F**: heatmap; Yellow represented high expression of regulators and blue represented low expression; **G**: Median expression of genes)

We demonstrated significantly differential pathways involved in metabolism between m6A-gene-clusters (Figure S1F). M6A-gene-cluster A exhibited a higher activity of hypoxia and glycolysis than m6A-gene-cluster B. The m6A-gene-cluster B was characteristic with higher activity of tricarboxylic acid cycle (Fig. 4A and B). Expression of FBP1, a key inhibitor of glycolysis [48], was significantly lower in m6A-gene-cluster A (Figure S3A). The correlation between median glycolytic expression and DEGs was showed in Table S3. MAPK13 was most strongly positively correlated with glycolysis (Spearman *r* = 0.44, *P* < 0.0001) and AQP9 was most strongly negatively correlated with glycolysis (Spearman *r*=-0.45, *P* < 0.0001) (Fig. 4C).

Expression of cholesterol metabolism related genes was higher in m6A-gene-cluster A (Fig. 4D). In addition, SOAT1 and SREBF2, two key regulators of cholesterol metabolism, were also significantly upregulated in m6A-gene-cluster A (Fig. 4E).M6A-gene-cluster A exhibited a higher expression of fatty acid biosynthetic process and a lower expression of fatty acid oxidation (Fig. 4F and G), causing fatty acid accumulation and promoting proliferation and migration of HCC cell.

In conclusion, m6A-gene-cluster A was characterized with higher activity of hypoxia, glycolysis, cholesterol metabolism, and fatty acid biosynthesis, while m6A-gene-cluster B exhibited higher activity of TCA (tricarboxylic acid cycle), fatty acid oxidation, steroid hormone metabolism (Figure S3B) and retinol metabolism (Figure S3C).

### Differential immune characteristics in distinct m6A modification patterns

**Fig. 5:**
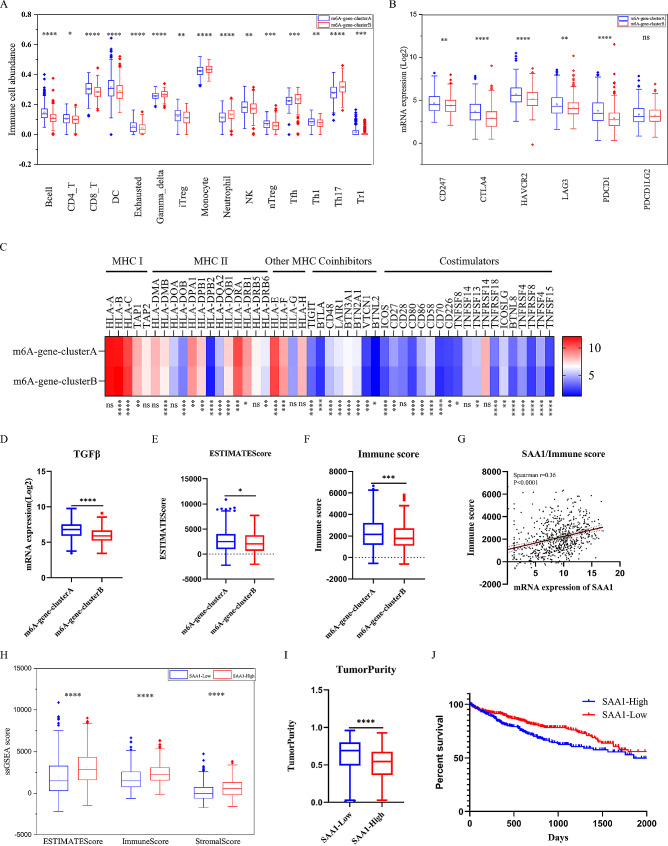
Differential immune characteristics in distinct m6A modification patterns. (**A**-**B**) The differential immune cell infiltration and the expression of 6 immune checkpoints between m6A-gene-cluster A and B. m6A-gene-cluster A, Grey; m6A-gene-cluster B, Red. The upper and lower ends of the boxes represented interquartile range of values. The lines in the boxes represented median value, and black dots showed outliers. The asterisks represented the statistical *p* value. (**C**) Comparison of the mRNA expression of the MHC molecules, co-stimulators and co-inhibitors for m6A-gene-clusters. (**D-F**) Comparison of the mRNA expression of TGFβ, ESTIMATE score and immune-score for m6A-gene-clusters. (**G**) Spearman correlation between SAA1 expression and immune score. (**H-I**) Comparison of ESTIMATE score, immune-score, stromal score and tumor purity between SAA1-High and SAA1-Low group. (**J**) Kaplan-Meier analysis for SAA1 expression in the TCGA cohort. (*P < 0.05; **P < 0.01; ***P < 0.001; ****P < 0.0001)

The m6A-gene-cluster A were remarkable with abundant B cell, CD8 T, Exhausted T, nTreg, Treg1 and higher expression of all immune checkpoints (Fig. 5A and B). Expression of most immune related genes was higher in m6A-gene-cluster A (Fig. 5C). However, expression of TGFβ was also higher in m6A-gene-cluster A (Fig. 5D), which was consistent with higher Treg abundance and expression immune checkpoints. Furthermore, ESTIMATE score and immune score of m6A-gene-cluster A were also higher than m6A-gene-cluster B (Fig. 5E, 5 F). Therefore, m6A-gene-cluster A was characterized as immune-activated and immune-suppressive simultaneously.

In correlation analysis between immune score and DEGs (Table S4), SAA1 (Serum Amyloid A), a protein associated with Amyloidosis, was found most positively correlated with immune score (Spearman *r* = 0.36, *P* < 0.0001) (Fig. 5G). In addition, HCCs with high expression of SAA1 exhibited higher immune score, stromal score, and Estimate score, indicating abundant immune infiltration and lower tumor purity (Fig. 5H and I). A better overall survival was showed in SAA1-High group (Fig. 5J). Above all, SAA1 might be a potential target for immune therapy.

### Characteristics of m6A score in prognosis, immune microenvironment and metabolism

**Fig. 6:**
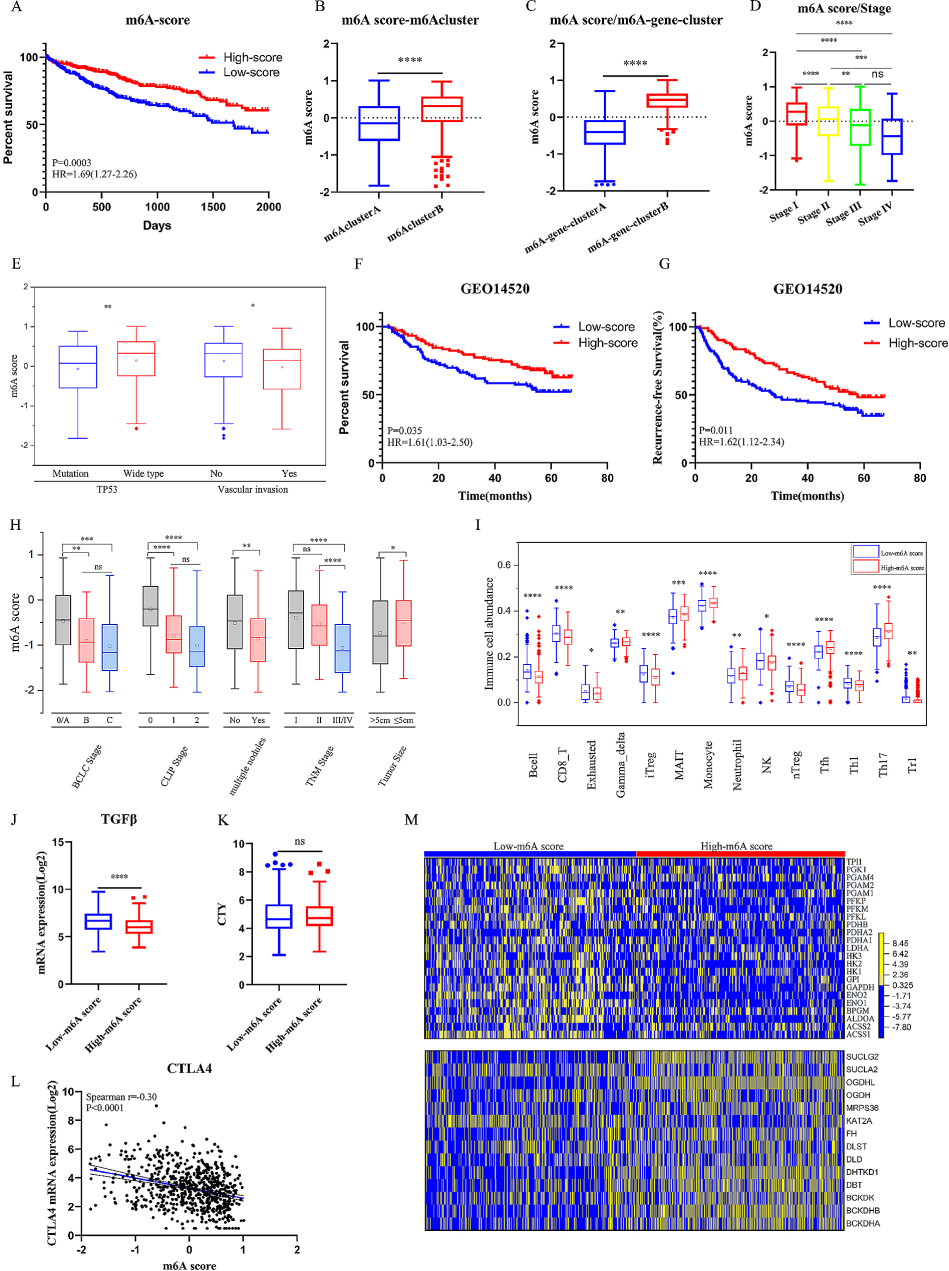
Characteristics of m6A score in prognosis, immune microenvironment and metabolism. (**A**) Survival plot of m6A score (High-m6A score group:335 HCCs; Low-m6A score group: 334 HCCs). The high-m6A score group showed better overall survival than the low- m6A score group (*P* = 0.0003; HR = 1.69). (**B**) Differences in m6Ascore between m6A-gene-clusters. The Mann-Whitney U test was used to compare the statistical difference between m6A-gene-clusters. High-m6A score group, Grey; Low-m6A score group, Red. The upper and lower ends of the boxes represented interquartile range of values. The lines in the boxes represented median value, and black dots showed outliers. The asterisks represented the statistical *p* value. (**C**) Differences in m6Ascore between m6A modification patterns (Mann-Whitney U test). (**D**) Differences in m6Ascore among different tumor stages (Mann-Whitney U test). (**E**) Differences in m6Ascore between TP53-mutation and TP53-wide type, vascular invasion and non-vascular invasion (Mann-Whitney U test). (**F**) Validation: Survival plot of m6A score in GSE14520 cohort. The high-m6A score group showed better recurrence-free survival than the low- m6A score group (*P* = 0.011; HR = 1.62). (**G**) Survival plot of m6A score in GSE14520 cohort. The high-m6A score group showed better overall survival than the low- m6A score group (*P* = 0.035; HR = 1.61). (**H**) Relationship between m6A score and TNM stage, BCLC stage, CLIP stage, tumor number, tumor size in validation cohort (GSE14520). (**I**) The differential immune cell infiltration between high-m6A score group and low-m6A score group. (**J, K**) Differences in expression of TGFβ and CYT (cytolytic activity) between high-m6A score group and low-m6A score group. (**L**) Spearman correlation between m6A score and expression of CTLA4. *P* < 0.0001. (**M**) Comparison of glucose metabolism related genes between high-m6A score group and low-m6A score group. (Heatmap; Yellow represented high expression of regulators and blue represented low expression). (*P < 0.05; **P < 0.01; ***P < 0.001; ****P < 0.0001)

In univariate analysis, prognostic factors associated with m6A related genes from DEGs was showed in Table S4. We constructed m6A score using principal component analysis in a cohort of 669 HCC patients. Survival analysis demonstrated patients with high-m6A score (Median m6A score = 0.11) had a better OS (*P* = 0.0003, HR = 1.69) (Fig. 6A). As expected, m6A score in m6Acluster B and m6A-gene-clusterB was significantly higher than m6Acluster A and m6A-gene-clusterA (Fig. 6B, 6 C). The m6A score was negatively correlated with TNM stage (AJCC, 2010) (Fig. 6D). In addition, HCCs with TP53 mutation and vascular invasion also had lower m6A score (Fig. 6E). In validated group of 225 HCC patients in GSE14520 cohort, high-m6A score was associated with better recurrence-free survival (RFS) (Fig. 6F) and overall survival (Fig. 6G). M6A score was associated with tumor stages in various staging systems, including TNM stage (AJCC, 2010), BCLC stage and CLIP stage in training cohort and validation cohort. HCCs with larger tumor size (> 5 cm) and multiple nodules also had lower m6A score (Fig. 6H).

In correlation between m6A score and tumor immune microenvironment, the low-m6A score group was characteristic with higher abundance of B cell, CD8 + T, NK, Th1, Exhausted, Treg1, nTreg, iTreg. However, the abundance of Gamma-delta T, MAIT, Monocyte, Neutrophil, Tfh and Th17 were higher in the high-m6A score group (Fig. 6I). TGFβ was significantly upregulated, indicating immune-inhibition in low-m6A score group (Fig. 6J). we also compared CYT between groups and found no differences (Fig. 6K). In addition, the m6A score was negatively correlated with expression of PDCD1 (Fig. 6L) and CTLA4 (Figure S5A). The stromal score was also lower in low-m6A score group (Figure S5B). The m6A score was positively correlated with Th17 and Tfh abundance, and negatively correlated with nTreg and B cell (Figure S5C).

In addition to the immune cell infiltration, we compared expression of glucose metabolism related genes between groups. Consistent with results of GSEA, GO and KEGG analysis, low-m6A score was correlated with significantly higher activity of glycolysis and lower activity of TCA (Fig. 6M). These characteristics could promote proliferation and migration of HCC and contribute to worse prognosis.

### Characteristics of m6A score in targeted therapy

**Fig. 7:**
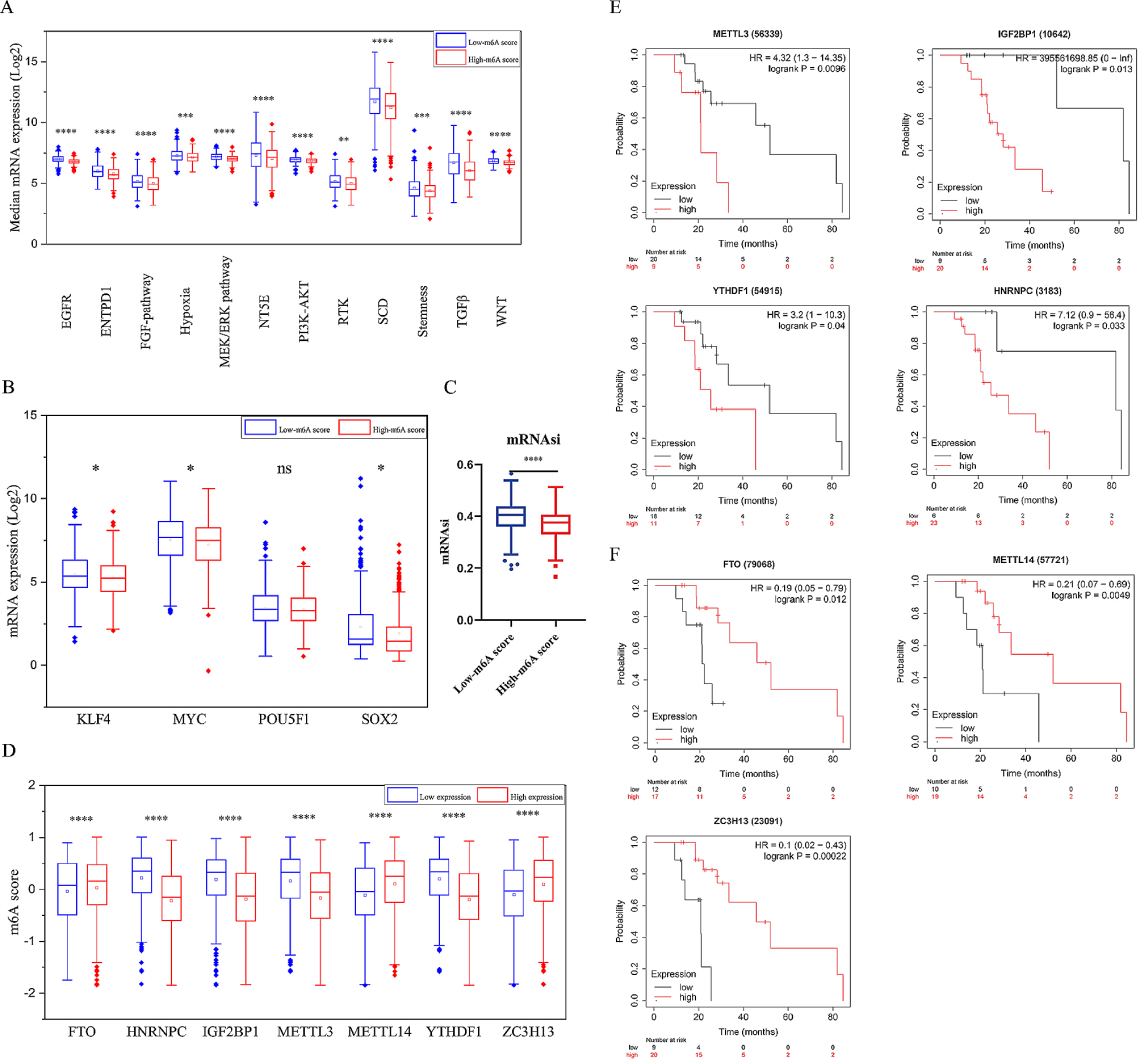
Characteristics of m6A score in targeted therapy resistance. (**A**) Differential median mRNA expression of targeted therapy resistance related signaling pathway between high-m6A score group and low-m6A score group. High-m6A score group, Grey; Low-m6A score group, Red. The upper and lower ends of the boxes represented interquartile range of values. The lines in the boxes represented median value, and black dots showed outliers. The asterisks represented the statistical *p* value. (**B**) Differences in expression of KLF4, OCT4, MYC and SOX2 between high-m6A score group and low-m6A score group. (**C**) Differences in mRNAsi between high-m6A score group and low-m6A score group. (**D**) Differences in m6Ascore between high expression group and low expression group of 6 m6A regulators (Mann-Whitney U test). (**E, F**) Differential overall survival between high expression group and low expression group of 6 m6A regulators in 29 HCCs who received Sorafenib therapy. (*P < 0.05; **P < 0.01; ***P < 0.001; ****P < 0.0001)

To examine whether m6A score was associated with targeted therapy and immunotherapy resistance, we compared mRNA expression of several pathways involved in drug resistance. Hypoxia [49], EGF-signaling [50], FGF-signaling [51], PI3K-AKT pathway [52], SCD [53] were associated with sorafenib resistance. In addition, TGFβ [54], hypoxia [55], stemness [56], WNT pathway [57], ENTPD1 and NT5E [58] were associated with immunotherapy resistance. In low-m6A score group, pathways involved in sorafenib resistance (hypoxia, EGF-signaling, FGF-signaling, MEK/ERK pathway, PI3K-AKT pathway, RTK signaling, SCD) and immunotherapy resistance (TGFβ, hypoxia, stemness, WNT pathway, ENTPD1 and NT5E) were significantly upregulated (Fig. 7A). We found differences in expression of KLF4, OCT4, MYC and SOX2 between groups (Fig. 7B). In addition, m6A score was negatively correlated with mRNAsi (Fig. 7C). Above all, m6A score was negatively correlated with stemness.

HCCs with low expression of HNRNPC, IGF2BP1, METTL3 and YTHDF1 had significantly higher m6A score, and low expression of FTO, METTL14 and ZC3H13 was associated with lower m6A score (Fig. 7D). In 29 HCC patients treated with Sorafenib from TCGA-LIHC cohort, patients with low expression of HNRNPC, IGF2BP1, METTL3 and YTHDF1, namely high m6A score, had a better overall survival (Fig. 7E). So did patients with high expression of FTO, METTL14 and ZC3H13 and high m6A score also had better overall survival (Fig. 7F).

## Discussion

Increasing evidences supported that m6A methylation plays a critical role in cancer. In this study, we uncovered different m6A modification patterns in HCC based on 23 m6A regulators. A total of 147 DEGs were identified between different m6A modification patterns (m6A-clusters). Notably, these m6A-gene-clusters exhibited remarkable differences in the tumor immune microenvironment and metabolism. Furthermore, we developed an m6A score based on the DEGs, which had the potential to predict prognosis and treatment response of targeted therapy in HCC (Figure S5).

Our findings showed that m6A modification serves as a novel regulator of metabolism. The GSEA of m6A modification patterns and pathway enrichment of DEGs were notably associated with various metabolic processes including glycolysis, TCA, cholesterol metabolism, fatty acid metabolism, steroid hormone metabolism and retinol metabolism. Accumulating evidence underscored the crucial role of metabolism in tumor formation, development, and progression, particularly in HCC [59]. Notably, cholesterol homeostasis has been implicated in the prognosis of HCC [60]. For instance, the knockdown of sterol O-acyltransferase 1 (SOAT1), a gene associated with high cholesterol levels, effectively suppressed the proliferation and migration of HCC [60]. In present study, we uncovered IGF2BP3, an effector protein of m6A modification, had strongest correlation with glycolysis and ENO1. Upregulation of IGF2BP3 enhanced activity of glycolysis and was associated with poorly prognosis. Previous studies have also confirmed the impact of metabolism-related pathways and metabolites on drug sensitivity [61, 62]. IGF2BP3 emerges as a potential target for metabolic treatments in HCC.

M6A modification has been proved to be a novel regulator of the immune system [63]. We found that the immune characteristics of m6A modification patterns in HCC were complicated. The m6A-gene-cluster A and low-m6A score group exhibited higher abundance of CD8 + T cells, NK cells, and B cells, which played a crucial role in antitumor immunity. Additionally, immune-related molecules and immune checkpoints were upregulated in this group, indicating potential sensitivity to immunotherapy. However, despite these immune features, both the m6A-gene-cluster A and low-m6A score group exhibited worse prognosis compared to the m6A-gene-cluster B and high-m6A score group. There are several reasons to consider for this observation. Firstly, regulatory T cells (nTreg, iTreg and Tr1) were also upregulated in the m6A-gene-cluster A and low-m6A score group. The high abundance of Treg cells in the TME has been associated with immune inhibition and worse prognosis [64]. Secondly, TGFβ, which was significantly upregulated in the m6A-gene-cluster A and low-m6A score group, has been linked to poor prognosis by promoting T-cell exclusion, inducing resistance to anti-PD1/PDL1 therapy, and facilitating immune evasion [54, 65]. In fact, the m6A-gene-cluster A and low-m6A score group exhibited characteristics of immune suppression, with antitumor immune cells such as B cell, CD8_T cell, NK cell, Th1 cell showing limited activation. Pathways such as PI3K-AKT [66], WNT [57, 67], hypoxia [55, 68], glycolysis, NT5E and ENTPD1 [58] have been reported to involved in immunotherapy resistance. As expected, the high-m6A score group showed sensitivity to anti-PD1 therapy and exhibited significant therapeutic advantages. For the m6Acluster A and low-m6A score group, a combination of TGFβ-blocking and anti-PD1/PDL1/CTLA4 therapy may facilitate antitumor immunity and enhance the sensitivity to immunotherapy. Many factors were found to be associated with targeted-therapy resistance, such as PI3K-AKT and JAK/STAT pathway [52], epithelial-mesenchymal transition [69], FGF-signaling [51], stemness [70], EGFR pathway [50], hypoxia [49] and fatty acid metabolism [53]. In our study, the activity of all these pathways and mRNAsi, an index represents stemness [42], were higher in low-m6A score group, which was consistent with outcome of sorafenib therapy.

Several limitations should be addressed in this study. The correlation between each m6A regulators and TME or metabolism was not fully explored. Further experiments are needed to explore the underlying regulatory mechanisms. Secondly, although we analyzed several immune cell types using bioinformatics approaches, our evaluation did not encompass all immune cell populations. Additionally, in this study, we combined TCGA-LIHC, GSE76427, ICGC-LIRI-JP data to obtain a larger cohort (669 HCCs, 292 normal) as the training cohort, and using the GSE14520 as the validating cohort. There might be the selection biases due to the small sample size. To strengthen the robustness of our findings, larger cohorts should be included in future. Moreover, there was no experimental validation of our findings in this study. Further experimental validation, like transcriptomic sequencing of HCC tissues, biological functional validation for m6A regulators and the 147 DEGs were worth exploring.

## Conclusion

We demonstrated the vital effect of m6A modification patterns and m6A regulators on TME, cancer metabolism and tumor development in HCC. Furthermore, we developed an m6A score that could effectively predict the response and outcomes of targeted therapy and immunotherapy. M6A regulators might be potential and promising targets for antitumor therapy of HCC.

### Electronic supplementary material

Below is the link to the electronic supplementary material.

Supplementary Material 1

Supplementary Material 2

Supplementary Material 3

Supplementary Material 4

Supplementary Material 5

## Data Availability

The datasets supporting the conclusions of this article are included within the article (and its additional file(s)).
