# Supplementary material for: Comprehensive analysis of m6A modification in immune infiltration, metabolism and drug resistance in hepatocellular carcinoma
Source: Cancer Cell Int. 2024 Apr 16;24:138. doi: 10.1186/s12935-024-03307-3 (PMC11022358; doi:10.1186/s12935-024-03307-3)
Supplement: Supplementary file 1 — Supplementary Material 1 [file 12935_2024_3307_MOESM1_ESM.docx]

**Supplementary Figures**


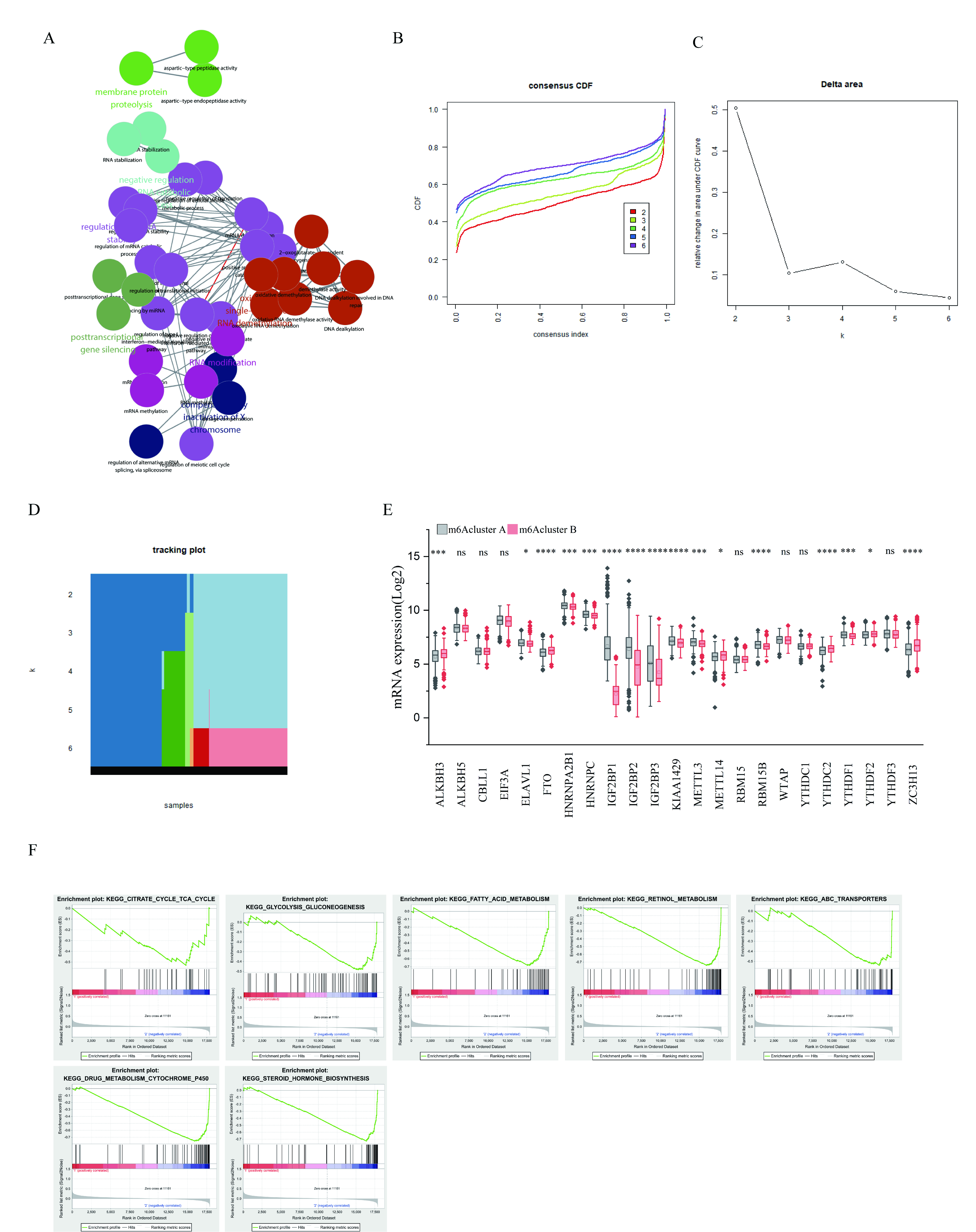


**Figure S1. Identification of m6A modification patterns.**

**(A)** GO analysis of 23 m6A regulators. **(B-D)** The CDF, Delta area and tracking plot of consensus. **(E)** Differential expression of 23 m6A regulators between m6Acluster A and m6Acluster B. m6Acluster A, Grey; m6Acluster B, Red. The upper and lower ends of the boxes represented interquartile range of values. The lines in the boxes represented median value, and black dots showed outliers. The asterisks represented the statistical p value. **(F)** GSEA analysis between m6Acluster A and m6Acluster B (P<0.05, FDR q<0.25), which showed significant difference in glucose metabolism, fatty acid metabolism, retinol metabolism and ABC transporters, drug metabolism and steroid hormone metabolism. (*P < 0.05; **P < 0.01; ***P < 0.001; ****P < 0.0001).


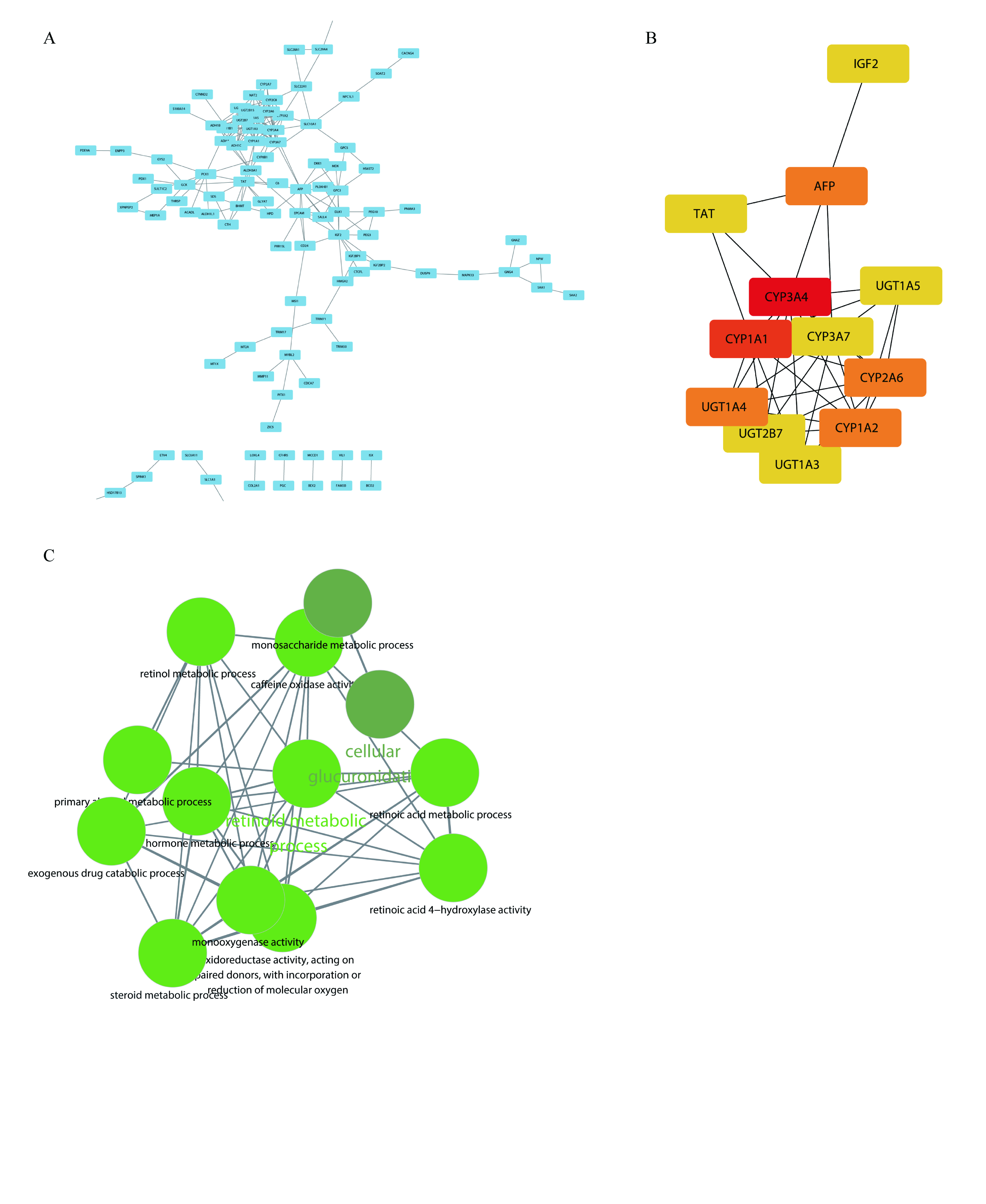


**Figure S2.** **Characteristics of 147 differential expression genes.**

**(A)** Protein-protein interaction network of DEGs. Rectangles represent protein. Edges represent protein-protein associations. **(B)** The top 12 hub genes ranked by degree in the PPI (Protein-protein interaction) network. Rectangles represent protein. Edges represent protein-protein associations. **(C)** GO analysis of the top 12 hub genes.


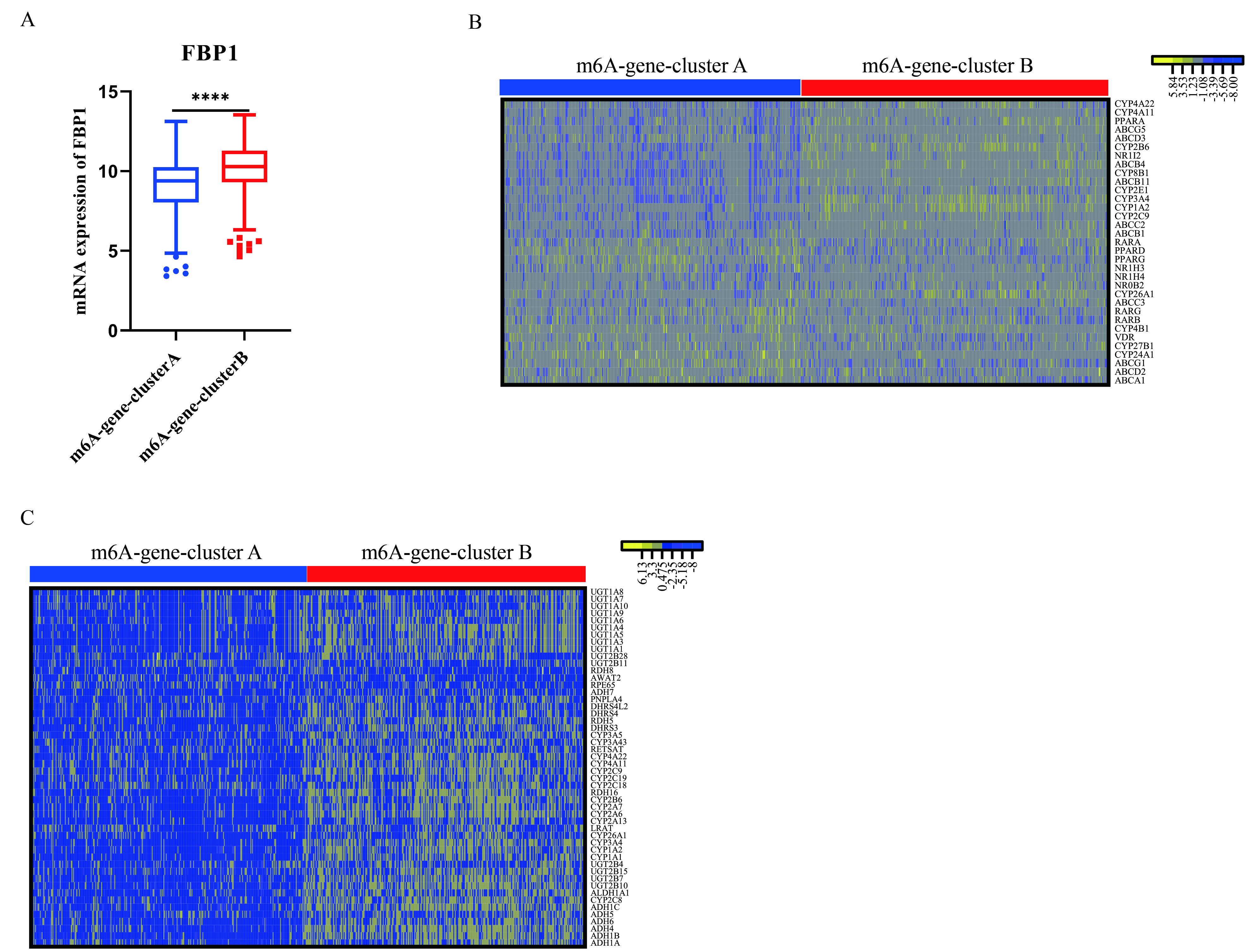


**Figure S3. Differential metabolic characteristics in distinct m6A modification patterns.**

**(A)** Differences in FBP1 expression between m6Acluster A and B. **(B)** Spearman correlation between IGF2BP3 and ENO1 expression. P<0.0001. Comparison of **(C, D)** steroid metabolism and retinol metabolism related genes between m6Acluster A and B. (heatmap; Yellow represented high expression of regulators and blue represented low expression). (*P < 0.05; **P < 0.01; ***P < 0.001; ****P < 0.0001).


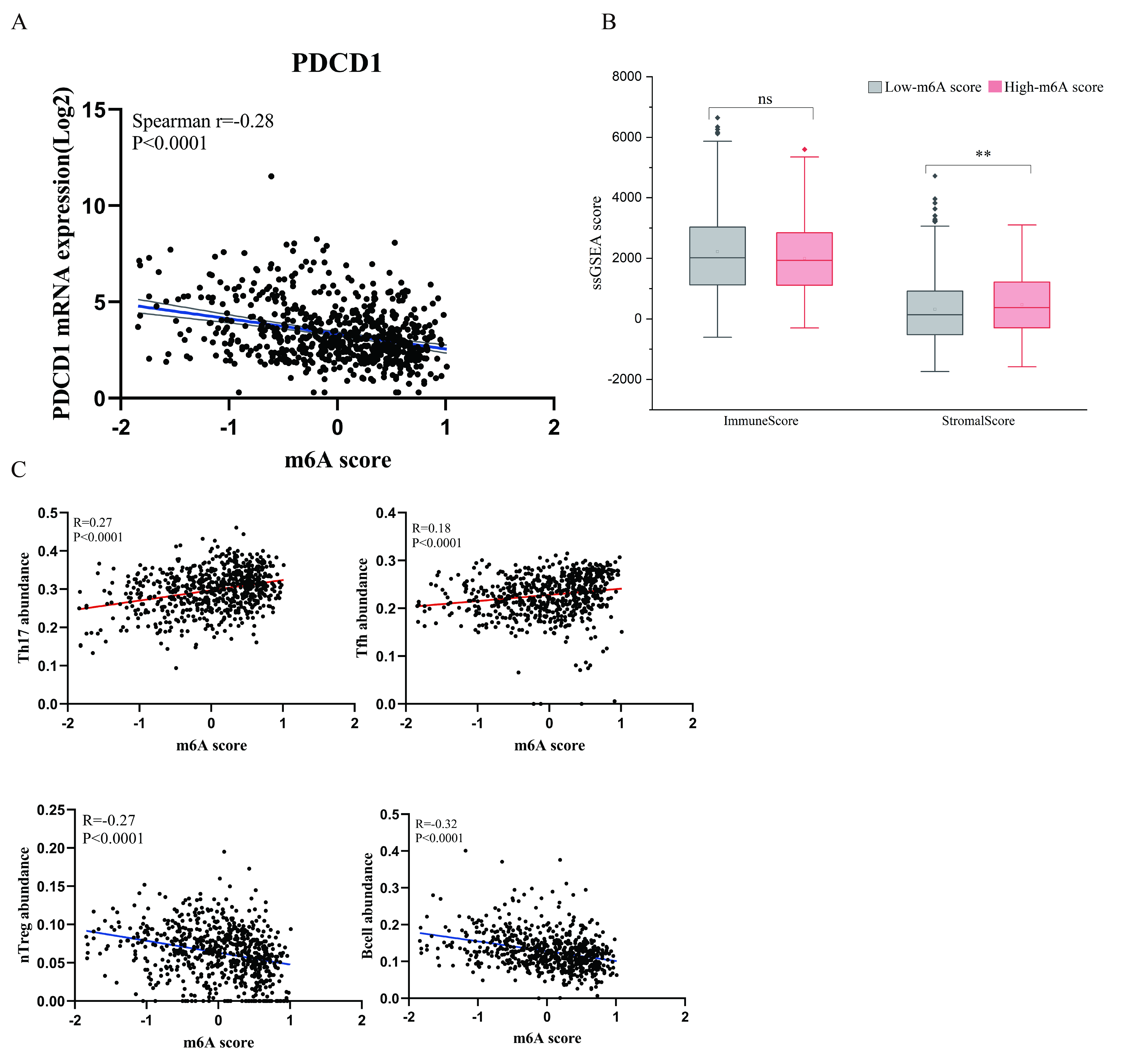


**Figure S4. Relationship between m6A score and immune characteristics.**

**(A)** Spearman correlation between m6A score and expression of PDCD1. P<0.0001. **(B)** Differences in immune-score and stromal-score between Low-m6A score and High-m6A score groups. (Mann-Whitney U test). Low-m6A score, Grey; High-m6A score, Red. The upper and lower ends of the boxes represented interquartile range of values. The lines in the boxes represented median value, and black dots showed outliers. The asterisks represented the statistical p value. **(C)** Spearman correlation between m6A score and immune cell abundance (Th17, Tfh, nTreg, Bcell). P<0.0001. (*P < 0.05; **P < 0.01; ***P < 0.001; ****P < 0.0001).


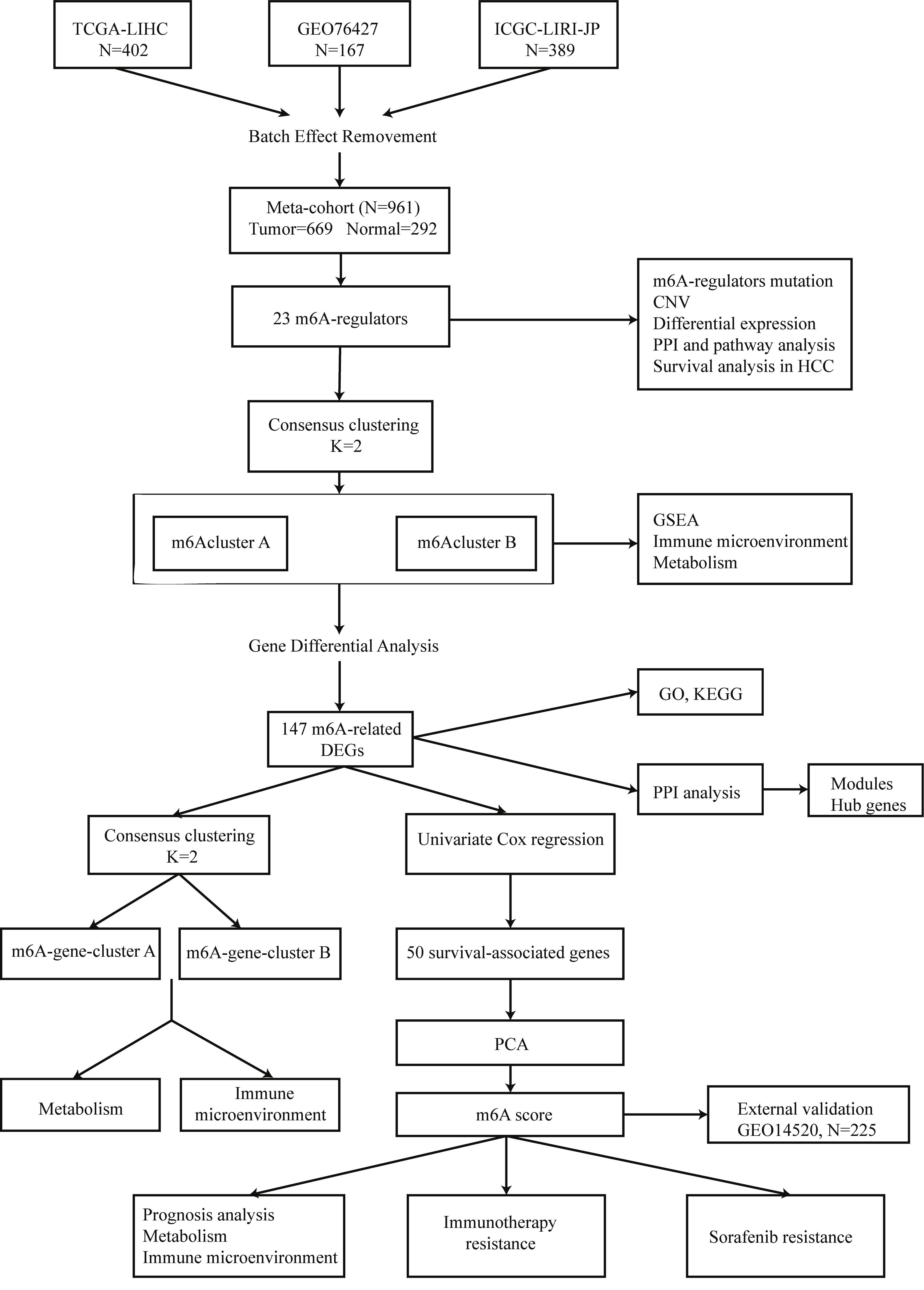


**Figure S5. Study flowchart.** 3 cohorts (TCGA-LIHC, GEO76427 and ICGC-LIRI-JP) were combined and 2 clusters were identified based on 23 m6A regulators. Characteristics in immune microenvironment and metabolism were comprehensively analyzed. We identified 147 DEGs between 2 clusters. 50 survival-associated genes were applied to construct m6A score. We also associated the m6A score with prognosis, targeted-therapy and immunotherapy resistance in HCC.

**Table S1. Correlation among 23 m6A Regulators (submitted as a separate Excel file).**

**Table S2. DEGs Between m6A clusterA and m6A clusterB (submitted as a separate Excel file).**

**Table S3. Correlation between 147 DEGs and glucose metabolism (submitted as a separate Excel file).**

**Table S4. Correlation Between 147 DEGs and Immunescore (submitted as a separate Excel file).**
